# Comparative efficacy of trastuzumab deruxtecan versus guideline-recommended treatments for 2L+ unresectable locally advanced or metastatic HER2-mutant non-small cell lung cancer: a systematic review and indirect treatment comparison

**DOI:** 10.3389/fonc.2025.1708245

**Published:** 2026-02-13

**Authors:** Federico Cappuzzo, Lirong Zhang, Kyle Dunton, Natalie Dennis, Pauline Le Nouveau, Agathe Nevière, Aline Gauthier

**Affiliations:** 1 National Cancer Institute Regina Elena, Rome, Italy; 2 Daiichi Sankyo Europe GmbH, Munich, Germany; 3 Daiichi Sankyo, Uxbridge, United Kingdom; 4 Daiichi Sankyo Oncology France, Rueil-Malmaison, France; 5 Amaris Consulting, Nantes, France; 6 Amaris Consulting, Barcelona, Spain

**Keywords:** HER2 mutation, matching-adjusted indirect comparison, network meta-analysis, NSCLC, trastuzumab deruxtecan

## Abstract

**Introduction:**

The clinical benefit of trastuzumab deruxtecan (T-DXd 5.4mg/kg), the first approved HER2-directed therapy for patients with previously treated HER2-mutant (HER2m) non-small cell lung cancer (NSCLC), was demonstrated in the phase II DESTINY-Lung02 trial. This study evaluated the efficacy of T-DXd relative to other approved treatments, including immunotherapies, vascular endothelial growth factor inhibitors, and chemotherapies, for adult patients with unresectable locally advanced or metastatic HER2m non-squamous NSCLC whose disease had progressed following ≥1 systemic therapy.

**Methods:**

A systematic literature review was conducted through September 2020 and supplemented in 2023 to identify relevant clinical trials. Given the single-intervention design in DESTINY-Lung02, two external comparator arms (ECAs) were created using docetaxel from INTEREST and VITAL, to connect T-DXd to a broader evidence network. Hazard ratios for progression-free survival (PFS) and overall survival (OS), and odds ratios (ORs) for overall response rate (ORR) were estimated via network meta-analysis. Matching adjusted indirect comparisons (MAICs) were also conducted for PFS and OS.

**Results:**

Fourteen studies with nine different regimens were included in the analysis. T-DXd showed better efficacy than all comparators, with a 100% probability of being the best treatment for PFS, ≥59% for OS, and ≥80% for ORR. Notably better PFS improvements were observed on T-DXd across all comparisons, with hazard ratios (HRs) [95% CrI] varying from 0.15 [0.09, 0.26] versus pemetrexed to 0.33 [0.20, 0.56] versus paclitaxel + bevacizumab. A similar trend was noted for OS. Patients on T-DXd maintained superior OS benefit versus other available treatments, with a notable difference demonstrated over paclitaxel + bevacizumab (HR [95% CrI]: 0.54 [0.30, 0.97]). As for ORR, the highest rate was achieved by T-DXd (49%), with odds ratios ranging from 6.09 to 21.14, representing a multifold increase compared with other regimens. Consistent results were obtained between the two different ECAs and the alternative approach via pairwise MAICs.

**Conclusion:**

This ITC suggested that T-DXd was associated with a consistent and meaningful benefit in terms of PFS and favorable OS relative to relevant comparators. For HER2m metastatic NSCLC adults, this review supports that T-DXd may be the best treatment option in the second-line or later settings.

## 1 Introduction

Lung cancer is the most common type of malignancy and the leading cause of cancer-related deaths globally, with approximately 2.48 million new cases and 1.82 million lung cancer-related deaths annually (1). Non-small cell lung cancer (NSCLC) is the largest subtype of lung cancer, representing 80-85% of all cases (2). Recently, the human epidermal growth factor receptor 2 (HER2 [*ERBB2]*) gene has been investigated as a primary oncogenic driver in NSCLC (3). Mutations in HER2 have been reported in 3% to 5% of NSCLCs (4–7), and 2% to 4% of lung adenocarcinomas (4–6, 8–10). Evidence suggests that HER2-mutant (HER2m) NSCLC patients have a poor prognosis and outcomes when treated with non-HER2 targeting agents and may have a higher incidence of central nervous system metastasis than HER2 wildtype patients (11).

In this era of personalized medicine, treatment of NSCLC typically is guided by the presence of oncogenic drivers and expression biomarkers, such as programmed cell death-ligand 1 (PD-L1). While the benefit of using targeted treatments in patients with driving mutations is well established, HER2 mutation was not regularly tested for in clinical practice prior to the availability of therapies targeted to this mutation, meaning that HER2m patients were largely managed similarly to other NSCLC patients who lack actionable molecular drivers. According to major guidelines, the recommended first-line therapy for these patients includes platinum-based chemotherapy (PBC), preferably with cisplatin, and immune checkpoint inhibitors (ICIs), given either as monotherapy or in combination (12–15). Their second-line therapy should then be driven by the treatments previously received and include immunotherapy agents, chemotherapy, or vascular endothelial growth factor (VEGF) inhibitors (12–14). These interventions, however, provide limited efficacy for HER2m NSCLC patients, offering a progression-free survival (PFS) benefit of only around 3–5 months and a median overall survival (OS) as short a 10 months or less (16–18).

Trastuzumab deruxtecan (T-DXd; Enhertu^®^) was the first HER2-directed therapy for patients with HER2m unresectable locally advanced or metastatic NSCLC (a/mNSCLC) in the second-line or later settings and is recommended by the European Society for Medical Oncology (ESMO) (19, 20), the National Comprehensive Cancer Network (NCCN) (21), and the American Society of Clinical Oncology (ASCO) guidelines (22). T-DXd is an antibody drug conjugate consisting of an anti-HER2 antibody and a novel topoisomerase I inhibitor linked by an enzymatically cleavable peptide linker. The efficacy and safety of T-DXd were evaluated in DESTINY-Lung01 (NCT03505710), a multicenter, open-label, phase II trial in which patients with HER2m NSCLC were treated with T-DXd at a dose of 6.4mg/kg every 3 weeks (Q3W) (23). Given the encouraging results, another multicenter, double-blind, randomized phase II trial, DESTINY-Lung02 (NCT04644237) (24), was subsequently initiated evaluating two different dosing regimens of T-DXd (5.4 mg/kg or 6.4 mg/kg Q3W) for the treatment of patients with HER2-mutant advanced or metastatic (a/m) NSCLC whose disease had progressed following one or more previous treatments, including platinum-based chemotherapy (PBC) (25). In both studies, T-DXd has demonstrated deep and durable responses in heavily previously treated a/mNSCLC patients, and considerable benefit in delaying progression and death. In addition, T-DXd has shown intracranial activity based on pooled data from DESTINY-Lung01 and DESTINY-Lung02 and the presence of brain metastasis at baseline did not affect systemic response (26). Given the favorable safety profile of the 5.4 mg/kg dose compared to the 6.4 mg/kg dose, the US Food and Drug Administration (FDA) and European Medicines Agency (EMA) approved T-DXd monotherapy at a dose of 5.4 mg/kg the treatment of adult patients with a/mNSCLC whose tumors have an activating HER2 mutation and who require systemic therapy following platinum-based chemotherapy with or without immunotherapy (27, 28).

Historically, patients who carry the HER2 mutation had limited treatment options, with no targeted therapies available. Following the T-DXd approval, guidelines from ESMO, NCCN, and ASCO now consider HER2 an actionable mutation and recommend T-DXd as the preferred treatment option for HER2m a/mNSCLC patients (19–22). Given the rarity of HER2m cancer, especially in the later line setting, to date, no clinical trials have directly compared T-DXd to other available therapies for the HER2m NSCLC patient population. The objective of this study was therefore to estimate the relative efficacy of T-DXd, based on the DESTINY-Lung02 trial, versus all relevant comparators for treatment of adults with a/mNSCLC whose disease had progressed following one or more systemic therapies. The indirect treatment comparison (ITC) was conducted in the non-squamous (NSQ) population following best practice guidelines to limit bias as all except one patient in the DESTINY-Lung02 trial had NSQ NSCLC. Moreover, around 70% of NSCLC patients have NSQ histology, with HER2 mutations being most prevalent in NSQ NSCLC (29).

## 2 Methods

### 2.1 Study selection

A systematic literature review (SLR) was conducted to identify clinical trials conducted in adults (≥18 years) with NSQ a/mNSCLC in accordance with National Institute for Health and Care Excellence (NICE) and Preferred Reporting Items for Systematic Reviews and Meta-Analyses (PRISMA) guidelines. The criteria for study selection for the ITC feasibility assessment are outlined in **Table 1**. Comparators of interest were selected based on the ASCO and ESMO guidelines for a/mNSCLC, market shares, and clinical expert opinion. See **Supplementary Materials** for details on the searches and study selection for the SLR. Descriptive statistics were used to assess similarity of the baseline characteristics of included studies with a focus on key prognostic factors and treatment effect modifiers (TEMs) identified through a targeted literature review (30–32) and validated by medical experts. The following factors were considered: age, sex, smoking status, disease stage (IIIB and IV), EGFR mutation status, other mutation status (e.g., ALK, KRAS), histology, Eastern Cooperative Oncology Group (ECOG)/World Health Organization (WHO) performance status, number of metastatic sites, previous treatment received, gross tumor volume, tumor diameter, nodal volume, and programmed death-ligand 1 (PD-L1) expression. Where possible, baseline characteristics from NSQ patients were used for heterogeneity assessment. If not available, patient characteristics for the mixed histology population were used instead. Because the majority of patients were NSQ, the mixed histology population was still considered to be representative. As HER2m status was not reported or tested in the comparator trials, it was not feasible to adjust for HER2 mutation. To address this issue, two steps were taken: 1) only studies conducted within patients who had wild type for other actionable driver mutations were included to ensure broadly comparable population, as in DESTINY-Lung02 patients who had a known driver mutation other than HER2m were excluded; 2) other characteristics reported to be correlated with HER2m status (including disease stage, smoking status, sex, age, and concurrent mutations) were considered and assessed carefully, to ensure they are well balanced amongst all included studies. Studies selected for the network meta-analysis (NMA) and matching-adjusted indirect comparison (MAIC) were based on sample size, overlap with the DESTINY-Lung02 population, and availability of prognostic factors and TEMs. For the MAIC, if several trials were eligible, the ones included in the NMA were preferred.

**Table 1:** Summary of the eligibility criteria for inclusion in the feasibility assessment of the ITC.

Component	Description
Population	Adult (≥18 years) patients with unresectable locally advanced or metastatic non-squamous NSCLC, regardless of HER2 gene mutation. The following specific criteria were used for the inclusion of studies: ◼ Studies including patients treated as 2^nd^ line or later-line ◼ Studies including non-squamous patients only or studies with both non-squamous and squamous patients but presenting stratified results by histology ◼ Studies on patients with wild-type onco-driver genes, or an undetermined or mixed mutation status for other established actionable genetic mutations (EGFR, ALK, ROS1, MET, KRAS)
Intervention	Trastuzumab deruxtecan (Enhertu^®^) 5.4 mg/kg
Comparators	◼ Platinum-based CT (i.e., including cisplatin, carboplatin, or oxaliplatin) ◼ Nivolumab (monotherapy) ◼ Atezolizumab (monotherapy) ◼ Pembrolizumab (monotherapy) for patients with PD-L1>1% ◼ Docetaxel ◼ Pemetrexed ◼ Ramucirumab in combination with docetaxel ◼ Nintedanib in combination with docetaxel ◼ Paclitaxel in combination with bevacizumab ◼ Other CT monotherapies (i.e., gemcitabine, vinorelbine, paclitaxel)
Outcomes	Efficacy outcomes: ◼ Progression-free survival (PFS) ◼ Overall survival (OS) ◼ Overall response rate (ORR)
Study design	Clinical trials (including single-arm trials)

ALK, Anaplastic lymphoma kinase; CT, Chemotherapy; DOR, duration of response; EGFR, Epidermal growth factor receptor; HER2, Human epidermal growth factor receptor 2; ITC, Indirect treatment comparison; KRAS, Kirsten rat sarcoma virus; NSCLC, Non-small cell lung cancer treatment; OS, Overall survival; PD-L1, Programmed death-ligand 1; PFS, Progression-free survival.

### 2.2 ITC

The NMA and MAIC were conducted following the National Institute for Health and Care Excellence (NICE) Decision Support Unit (DSU) guidelines (33, 34) and using the primary data cut-off (23 December 2022) for DESTINY-Lung02. See **Supplementary Materials** for a detailed description of the NMA and MAIC methods.

#### 2.2.1 Construction of an external comparator arm and network meta-analysis

To overcome the lack of connectivity, an external comparator arm (ECA) was first generated to bring the T-DXd 5.4 mg/kg treatment arm from DESTINY-Lung02 into the network of available evidence. Patient-level data from two historical clinical trials in NSCLC were utilized, and key patient characteristics were matched to DESTINY-Lung02 via propensity score weighting. Propensity score weighting was used to balance observed baseline characteristics to make patients in the ECA more comparable to patients enrolled in DESTINY-Lung02. The ECA was assessed by reviewing the effective sample size (ESS) and the distribution of the propensity score weights by treatment arm. Descriptive statistics were performed for patient characteristics that were identified as prognostic factors and TEMs, within the reweighted populations, to assess whether substantial differences remained between the groups.

An NMA was subsequently conducted in a Bayesian framework on the connected network for PFS, OS, and overall response rate (ORR). All outcome data were from the NSQ population of the respective trial. NMA results were expressed as median and associated 95% credible interval (CrI) of the posterior distribution of the relative treatment effect (hazard ratio [HR] for PFS and OS, odds ratio [OR] for ORR) for all pairwise comparisons. Results were considered to be notably different if the 95% CrI did not cross 1. The pairwise probabilities for T-DXd 5.4 mg/kg to perform better than each comparator of interest were also generated (35).

#### 2.2.2 Matching-adjusted indirect comparisons

Unanchored MAICs were conducted to compare PFS and OS outcomes for T-DXd 5.4 mg/kg with each relevant comparator. Variables identified as prognostic factors and/or TEMs were used in the weighting process. See **Supplementary Materials** for further details. Results were considered significant when the confidence interval (CI) did not include 1. Following the availability of data from a later cut-off for DESTINY-Lung02 (25 August 2023), additional MAICs were conducted as a scenario analysis.

## 3 Results

### 3.1 Study selection and heterogeneity assessment

A total of 91 papers were identified via the SLR and hand searches, corresponding to 70 clinical trials (**Supplementary Figure S1**). After applying the ITC PICOS criteria (**Table 1**), 18 randomized controlled trials (RCTs) including at least one outcome of interest for NSQ patients were identified for consideration in the feasibility assessment. Five single-arm trials with treatments of interest were also considered for the MAICs; however, they were ultimately not used in favor of RCTs. Eight RCTs were conducted in an entirely NSQ population, and the remaining ten in a mixed histology population. All prognostic factors and TEMs were assessed across the trials, including age, sex, prior lines of therapy, smoking status, disease stage, ECOG/WHO performance status, EGFR mutation, and adenocarcinoma patients. Based on the findings of the feasibility assessment, 13 trials were included for comparison with DESTINY-Lung02 in the NMAs and MAICs. Overall, the included trials were well balanced, with some differences observed for certain characteristics, including the proportion of males and the proportion of patients who never smoked (**Table 2**). Trials excluded from both the NMA and MAIC are listed along with the rationale for exclusion in **Supplementary Table S1**.

**Table 2:** Baseline characteristics and analyses conducted for RCTs included in the NMA and MAIC.

Trial (population)	Baseline characteristics in the NSQ or ITT population										Analysis conducted	
	% NSQ	Mutation status	Age (median, years)	Males	Prior lines of therapy	Smoking status	Disease stage	ECOG performance status	Adeno- carcinoma	Subsequent therapy	NMA	MAIC
T-DXd 5.4 mg/kg												
DESTINY-Lung02 (NSQ)	99%	NR	58	36%	≤2: 68% >2: 32%	Ever: 46% Never: 54%	Stage IV: 100%	PS 1: 71% PS 0: 29%	98%	32.5%	PFS, OS, ORR	PFS, OS
Docetaxel 75 mg/m^2^												
KEYNOTE-010 (ITT)	70%	EGFrm (6%) ALKt (1%)	62	61%	Prior PLT	NR	NR*	PS 1: 65% PS 0: 34%	NR	44.0%	PFS, OS	N/A
CheckMate 057 (NSQ)	100%	EGFRm (13-15%) ALKt (3-4%) KRASm(10-12%)	64	58%	1: 89% 2: 11%	Ever: 78% Never: 21%	Stage IIIB: 8% Stage IV: 92%	PS 1: 67% PS 0: 33%	NR	52.0%	PFS, OS, ORR	PFS, OS
CheckMate 078 (ITT)	60%	EGFRm (0%)	60	81%	Prior PLT	NR	NR*	PS 1: 87% PS 0: 13%	NR	49.0%	PFS, OS, ORR	N/A
OAK (ITT)	74%	EGFRm (9%) KRASm (8%) EML4-ALKt (<1%)	64	62%	1: 76% 2: 24%	NR	NR	PS 1: 62% PS 0: 38%	NR	47.3%	OS	N/A
POPLAR (ITT)	66%	EGFRm (10-12%) KRASm (43-33%)	62	53%	Prior CT	NR	NR*	PS 1: 32% PS 0: 68%	NR	41.3%	OS	N/A
REVEL (ITT)	72%	EGFRm (3%)	61	66%	1: 100%	NR	NR*	PS 1: 68% PS 0: 32%	NR	54.9%	PFS, OS, ORR	N/A
IFCT-1103 ULTIMATE (NSQ)	100%	EGFRm (0%)	60	76%	1: 71% 2: 26% 3: 4%	Never: 16.4%	NR	PS 2: 93% PS 0 or 1: 7%	93%	38.2%	PFS, OS, ORR	PFS, OS
SIGN (NSQ)	100%	NR	60	70%	>2: 99%	Ever: 67% Never: 25% Unknown: 8%	NR	PS ≥2: 37% PS 1: 44% PS 0: 19%	NR	NR	PFS, OS, ORR	N/A
TAILOR (NSQ)	72%	EGFRm (0%)	67	66%	1: 100%	NR	NR*	PS 2: 6-8% PS 1: 44-45% PS 0: 48%	NR	N/A	PFS	N/A
LUME- Lung 1 (NSQ)	51%	EGFRm (1.6-3.3%)	59 (mean)	62%	>2: 100%	Ever: 66% Never: 34%	NR	PS 1: 70% PS 0: 30%	100%	53.8%	PFS, OS, ORR	N/A
Docetaxel 60 mg/m^2^												
DELTA (ITT)	79%	EGFRm (22%)	67	71%	2: 15%	NR	Stage IIIB: 19% Stage IV: 81%	NR	NR	N/A	PFS	N/A
Pembrolizumab												
KEYNOTE-010 (ITT)	70%	EGFrm (8%) ALKt (1%)	63	62%	Prior PLT	NR	NR*	PS 1: 65-67% PS 0: 33-35%	NR	NR	PFS, OS	N/A
Nivolumab												
CheckMate 057 (NSQ)	100%	EGFRm (13-15%) ALKt (3-4%) KRASm(10-12%)	61	52%	1: 88% 2: 12%	Ever: 79% Never: 20%	Stage IIIB: 7% Stage IV: 93%	PS 1: 71% PS 0: 29%	NR	46%	PFS, OS, ORR	PFS, OS
CheckMate 078 (ITT)	61%	EGFRm (0%)	60	78%	Prior PLT	NR	NR	PS 1: 86% PS 0: 14%	NR	49%	PFS, OS, ORR	N/A
Atezolizumab												
OAK (ITT)	74%	EGFRm (10%) KRASm (7%) EML4-ALKt (1%)	63	62%	1: 76% 2: 24%	Never: 18.3%	NR*	PS 1: 64% PS 0: 36%	NR	51.8%	OS	N/A
POPLAR (ITT)	66%	EGFRm (1%) KRASm (33%)	62	65%	Prior CT	Never: 18.8%	NR*	PS 1: 32% PS 0: 68%	NR	40.3%	OS	N/A
Ramucirumab + docetaxel												
REVEL (ITT)	74%	EGFRm (2%)	62	67%	1: 100%	Non-smoker: 17.4%	NR*	PS 1: 67% PS 0: 33%	NR	51.0%	PFS, OS, ORR	N/A
Paclitaxel + bevacizumab												
IFCT-1103 ULTIMATE (NSQ)	100%	EGFRm (0%)	60	70%	1: 68% 2: 31% 3: 1%	Never: 8.1%	NR	PS 2: 93% PS 0 or 1: 7%	90%	58.7%	PFS, OS, ORR	PFS, OS
Pemetrexed												
HORG (ITT)	78%	EGFRm (8%)** KRASm (29%)**	66	83%	2: 61% 3: 39%	NR	Stage IIIB: 11% Stage IV: 87%	NR	NR	N/A	PFS	N/A
CTONG0806 (NSQ)	100%	NR	58 (mean)	62%	NR	Ever: 42% Never: 58%	Stage IIIB: 13% Stage IV: 87%	PS 1: 82% PS 0: 18%	95%	98.7%	PFS, OS, ORR	PFS, OS
Nintedanib + docetaxel												
LUME- Lung 1 (NSQ)	49%	EGFRm (1.6-3.3%)	58 (mean)	63%	>2: 100%	Ever: 64% Never: 36%	NR	PS 1: 70% PS 0: 30%	100%	58.1%	PFS, OS, ORR	N/A

*The population included in the study had advanced or metastatic NSCLC.

**The percentages were taken from patients how had adequate tumor tissue available with a total sample size of 123 for EGFRm and 109 for KRASm.

ALKt, anaplastic lymphoma kinase translocated; CT, chemotherapy; ECOG, Eastern Cooperative Oncology Group; EGFR, epidermal growth factor receptor; ITT, intent-to-treat; KRASm, kirsten rat sarcoma virus mutated; MAIC, matching-adjusted indirect comparison; N/A, not applicable; NMA, network meta-analysis; NR, not reported; NSQ, non-squamous; ORR, overall response rate; OS, overall survival; PFS, progression-free survival; PLT, platinum; PS, performance score.

### 3.2 Network meta-analysis

To connect T-DXd 5.4 mg/kg from DESTINY-Lung02 to the network, an ECA was generated using patient-level data from previous clinical trials. The docetaxel arm from the INTEREST trial (36) was selected for the base case analysis due to better alignment of the population with DESTINY-Lung02 in terms of the number of prior therapies. The docetaxel arm from the VITAL trial (37) was used as a sensitivity analysis, as most patients in this trial had one prior lines of therapy (98.3%) compared to only 29.5% for T-DXd from DESTINY-Lung02. Inclusion and exclusion criteria from DESTINY-Lung02 were applied, and propensity score weighting was used to create an ECA with comparable prognostic factors to patients enrolled in DESTINY-Lung02. Overall, the patient characteristics were balanced between trials after weighting, except the prevalence of brain metastasis, which remained higher in the T-DXd 5.4 mg/kg treatment arm from DESTINY-Lung02 (38.6%) compared to INTEREST (17.2% after weighting). The proportions of patients who received at least 2 prior regimens was also higher in the T-DXd 5.4 mg/kg treatment arm (70.5%) compared to docetaxel 75 mg/m^2^ from VITAL (1.4% after weighting) (**Supplementary Table S2**).

**Figure 1:**
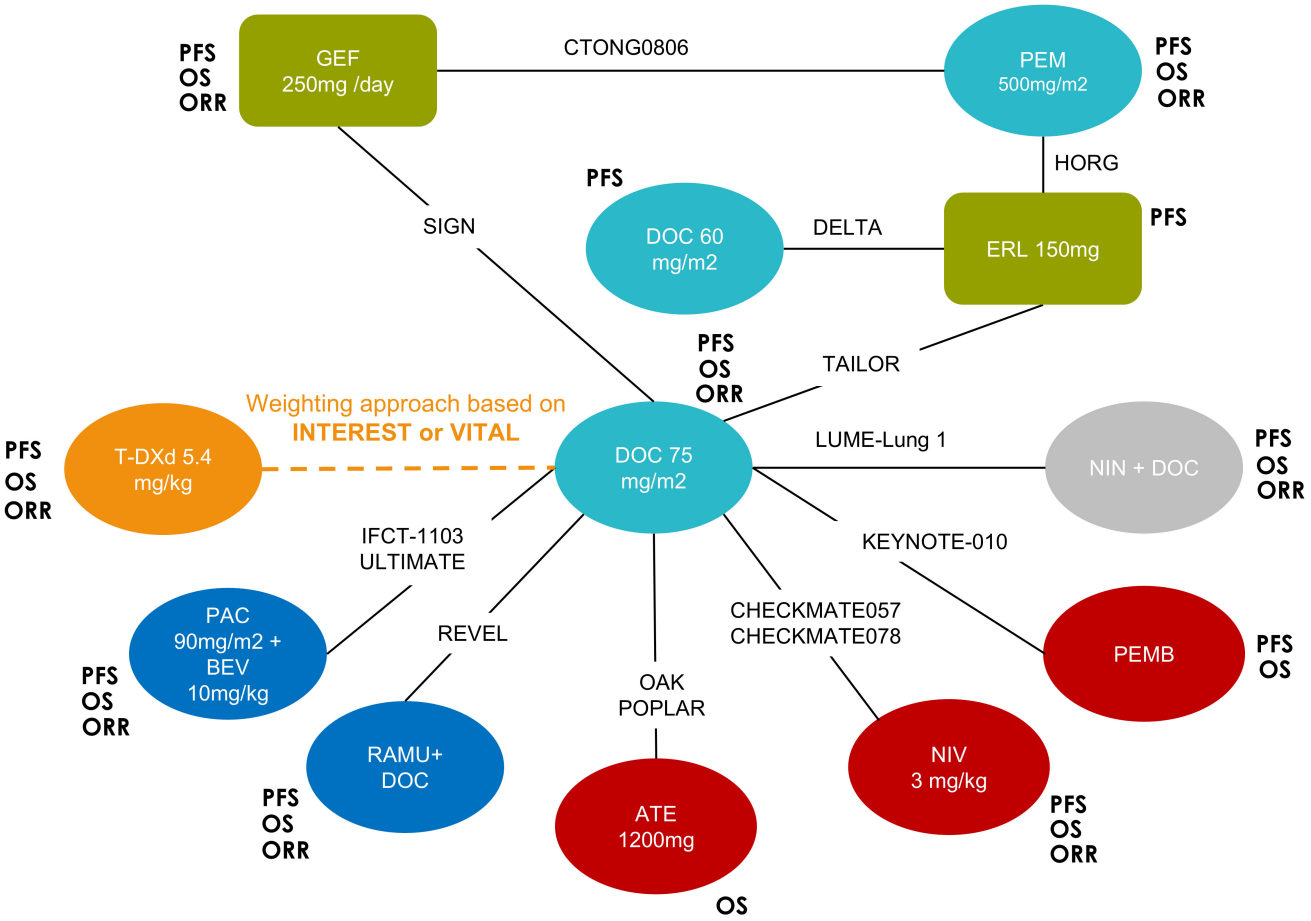
Global network of evidence for all three outcomes. The outcomes for which the comparator was included in the network is indicated in bold. Chemotherapies are indicated in turquoise, immunotherapies in red, tyrosine kinase inhibitors in green, vascular endothelial growth factor-targeted therapies in dark blue, and non-EGFR-targeted TKIs in grey. ATE, atezolizumab; BEV, bevacizumab; DOC, docetaxel; ERL, erlotinib; EGFR, epidermal growth factor receptor; GEF, gefitinib; NIN, nintedanib; NIV, nivolumab; ORR, overall response rate; OS, overall survival; PAC, paclitaxel; PEM, pemetrexed; PEMB, pembrolizumab; PFS, progression-free survival; RAMU, ramucirumab.

The global network of evidence for all three outcomes (PFS, OS, and ORR) is presented in **Figure 1**, with T-DXd 5.4 mg/kg connected via the docetaxel ECA. A total of 12 trials were included for PFS, 11 for OS, and 8 for ORR. Gefitinib and erlotinib were included to allow connectivity of the network; however, they are not comparators of interest, and their results are not presented. Inconsistency was initially found in the loop identified in the PFS network among gefitinib, pemetrexed, erlotinib, and docetaxel 75 mg/m^2^ (**Supplementary Table S3**), and two trials (SIGN and CTONG0806) were subsequently excluded from the PFS network based on their shorter length of follow-up (9 months for SIGN and 10.4 months for CTONG0806) (38, 39). Some violations were found with the PHA (**Supplementary Table S4**); however, given the star-shaped network, violation of the PHA for one comparison does not impact the validity of the results for the other comparisons (40).

**Figure 2:**
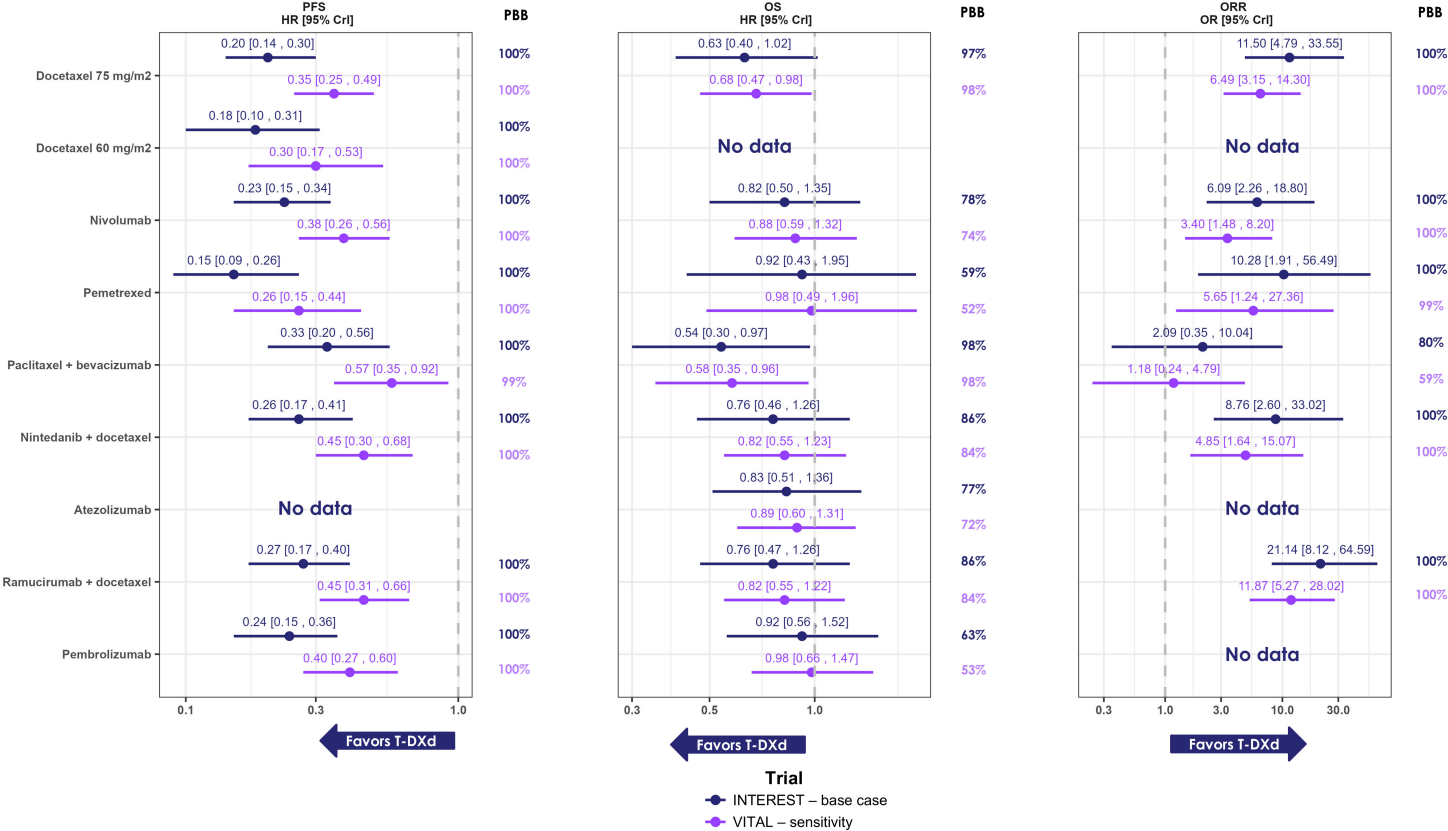
Forest plots of results obtained through the NMA for PFS, OS, and ORR. Note: gefitinib and erlotinib were included in the network; however, they were not comparators of interest and the results are not shown here. Results are considered notably in favor of T-DXd for PFS and OS when the HR is <1 and 95% CrI does not cross 1, and for ORR when the OR is >1 and the 95% CrI does not cross 1. CrI, credible Interval; OR, odds ratio; ORR, overall response rate; PBB, probability of being the best; T-DXd, trastuzumab deruxtecan.

T-DXd 5.4 mg/kg was associated with longer PFS when compared to all other treatments, with a 100% probability of being better than each comparator (**Figure 2**). T-DXd was found to be notably better than all comparators with HRs (95% CrI) ranging from 0.15 (0.09–0.26) versus pemetrexed to 0.33 (0.20–0.56) versus paclitaxel + bevacizumab. The sensitivity analysis including SIGN and CTONG0806 showed similar results (**Supplementary Table S5**; see **Supplementary Materials** for more details) (38, 39). A similar trend was observed for OS. The probability of T-DXd 5.4 mg/kg providing better OS than all comparators was the highest compared to paclitaxel + bevacizumab (97.94%) and docetaxel 75 mg/kg (97.11%), and at the lowest when compared to pemetrexed (59.01%) (**Figure 2**). T-DXd 5.4 mg/kg was found to be associated with a notably longer OS compared to paclitaxel + bevacizumab (HR [95% CrI]: 0.54 [0.30–0.97]). For the remaining comparators, OS was numerically in favor of T-DXd 5.4 mg/kg (HRs [95% CI] ranging from 0.63 [0.40, 1.02] to 0.92 [0.43, 1.95]).

Regarding ORR, T-DXd 5.4 mg/kg was associated with the highest response rate, with ≥80% probability being better than all comparators of (**Figure 2**). T-DXd 5.4 mg/kg was found to have a notably better ORR than most comparators: docetaxel 75 mg/m² (OR [95% CrI]: 11.50 [4.79, 33.55]), pemetrexed (OR [95% CrI]: 10.28 [1.91, 56.49]), nivolumab (OR [95% CrI]: 6.09 [2.26, 18.80]), ramucirumab + docetaxel (OR [95% CrI]: 21.14 [8.12, 64.59]), and nintedanib + docetaxel (OR [95% CrI]: 8.76 [2.60, 33.02]).

Results from the sensitivity analysis using VITAL to generate the ECA were overall aligned with the base case scenario (**Figure 2**). For PFS, T-DXd was found to be notably better than all other comparators when using the VITAL trial to link the network, with HRs [95% CrI] varying from 0.26 [0.15, 0.44] versus pemetrexed to 0.57 [0.36, 0.92] versus paclitaxel + bevacizumab. For OS, T-DXd was found to be notably better when compared to paclitaxel + bevacizumab (HR [95% CrI]: 0.58 [0.35, 0.96]) and docetaxel 75 mg/m^2^ (HR [95% CrI]: 0.68 [0.47, 0.98]). For ORR, T-DXd was found to have a notably better ORR when compared to docetaxel 75 mg/m^2^, pemetrexed, nivolumab, ramucirumab + docetaxel, and nintedanib + docetaxel with ORs [95% CrI] varying from 3.40 [1.48, 8.2] to 11.87 [5.27, 28.02].

### 3.3 MAICs

Seven MAICs were conducted for OS and PFS (**Table 2**). Comparator trials included second- or third-line patients and higher proportions of males and smokers. Patients from the DESTINY-Lung02 T-DXd 5.4 mg/kg arm were restricted to reflect the inclusion criteria of comparator trials and were adjusted for the following prognostic factors and TEMs where available: age, sex, smoking status, number of prior treatment lines of systemic therapy, ECOG/WHO score, and metastatic sites. The ESS ranged from 25.2 to 30.7. The PHA was generally considered to be valid for the majority of trials, except for the OS analysis of T-DXd versus nintedanib + docetaxel and pemetrexed (**Supplementary Table S6**).

**Figure 3:**
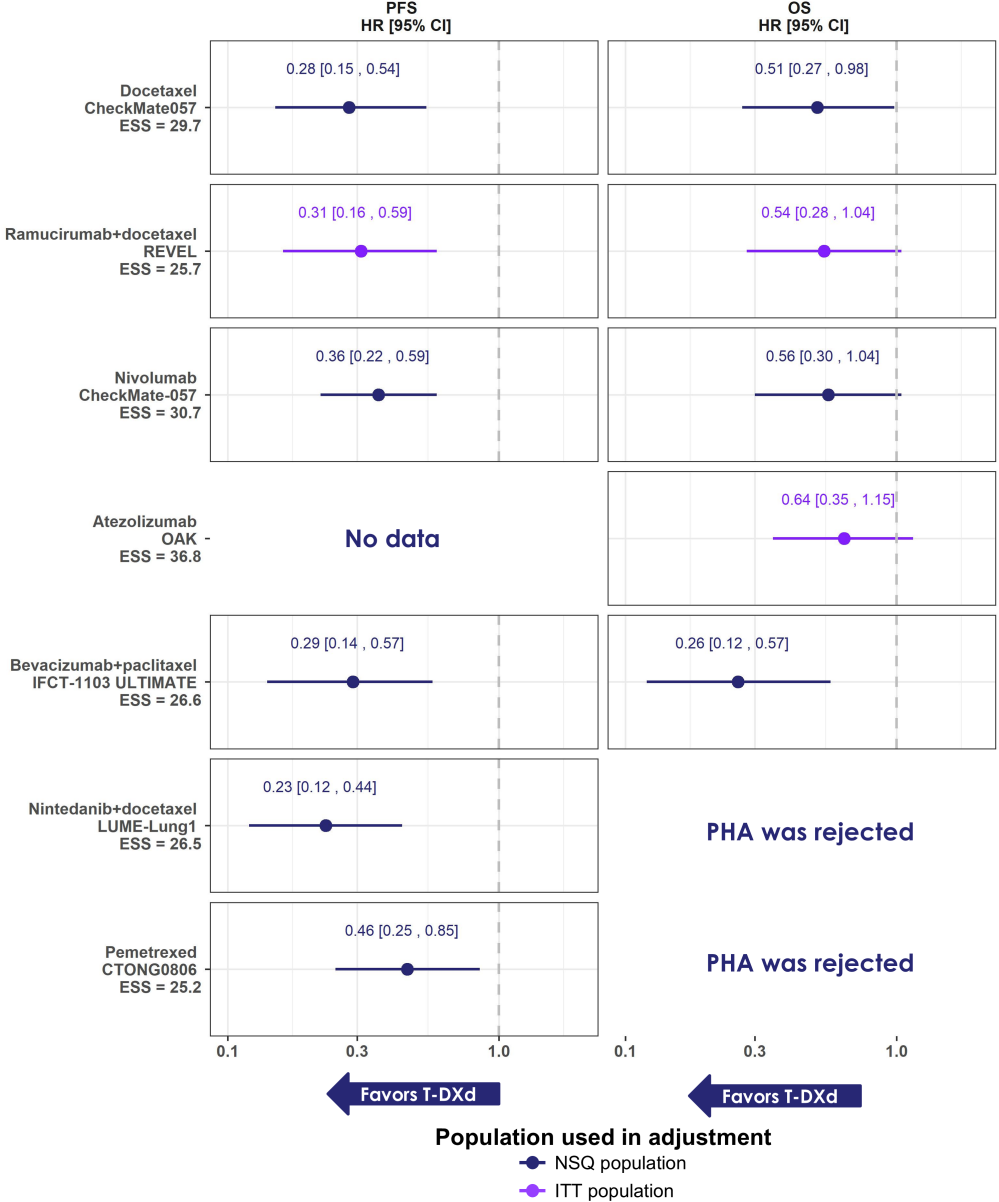
Summary forest plots of MAIC results obtained for PFS and OS, HR [95% CI] T-DXd versus comparator. Note: estimates based on NSQ population are indicated in blue, estimates based on ITT population are indicated in purple. Comparison versus pembrolizumab via unanchored MAIC was infeasible due to the lack of Kaplan-Meier data for pembrolizumab in the NSQ population. Results are considered significantly in favor of T-DXd when the HR is <1 and CI does not cross 1, and for ORR when the OR is >1 and the 95% CrI does not cross 1. Cl, confidence interval; HR, hazard ratio; ITT, intent-to-treat; MAIC, matching-adjusted indirect comparison; NSQ, non-squamous; OS, overall survival; PHA, proportional hazard assumption; PFS, progression-free survival; T-DXd, trastuzumab deruxtecan; ESS, effective sample size.

T-DXd 5.4 mg/kg demonstrated significantly better results across all comparisons for PFS (HRs [95% CI] ranging from 0.23 [0.12; 0.44] to 0.46 [0.25, 0.85]) (**Figure 3**). Similarly, the results were numerically in favor of T-DXd 5.4 mg/kg versus all comparators for OS (HRs [95% CI] ranging from 0.26 [0.12; 0.57] to 0.64 [0.35, 1.15]), and significantly better for T-DXd 5.4 mg/kg versus docetaxel (HR [95% CI]: 0.51 [0.27, 0.98]) and paclitaxel + bevacizumab (HR [95% CI]: 0.26 [0.12, 0.57]).

Sensitivity analyses conducted on the Full Analysis Set of DESTINY-Lung02 showed similar results as the base case MAICs (**Supplementary Table S7**; see **Supplementary Materials** for more details). In addition, the results of the sensitivity analysis using the later cut-off of DESTINY-Lung02 (25 August 2023) were consistent with those based on the primary data cut-off (**Supplementary Figure S2**; see **Supplementary Materials** for more details). Results for PFS suggest that T-DXd significantly reduces the risk of progression or death compared with all comparators, with the August 2023 data cut-off comparisons providing slightly narrower 95% CIs. Results for OS suggest that T-DXd improves OS for all comparators, with a statistically significant difference demonstrated over docetaxel, bevacizumab + paclitaxel, nivolumab, and ramucirumab + paclitaxel.

## 4 Discussion

To our knowledge, this is the first indirect treatment comparison to estimate the relative efficacy of T-DXd 5.4 mg/kg to other available therapies for the treatment of adults with HER2m, NSQ a/mNSCLC whose disease had progressed following one or more systemic therapies. Comparator trials were identified based on a comprehensive SLR and evaluated based on their outcomes of interest, relevance of the comparator, and similarity of baseline patient characteristics to the DESTINY-Lung02 trial. Following best practice guidelines, an ECA was generated using the docetaxel arms of the INTEREST trial in the base case and the VITAL trial as a sensitivity analysis to connect the T-DXd 5.4 mg/kg treatment arm from DESTINY-Lung02 to the network of available evidence, after which an NMA was conducted. MAICs were also conducted to ensure a comprehensive comparative assessment.

Patients treated with T-DXd 5.4 mg/kg were found to have longer PFS than all comparators in the base case NMA, with the greatest improvements seen over chemotherapies (docetaxel 75 mg/m^2^, 60 mg/m^2^, and pemetrexed; HRs [95% CrI] ranging between 0.15 [0.09, 0.26] and 0.20 [0.14, 0.30]) and immunotherapies (nivolumab and pembrolizumab; HRs [95% CrI] ranging between 0.23 [0.15, 0.34] and 0.24 [0.15, 0.36]). In addition, T-DXd showed an overall benefit for OS, with a notably longer OS than paclitaxel + bevacizumab (HR [95% CrI]: 0.54 [0.30, 0.97]). Greater improvements in OS were seen for T-DXd 5.4 mg/kg compared with VEGF-targeted therapies (paclitaxel + bevacizumab and ramucirumab + docetaxel; HRs [95% CrI] of 0.54 [0.30, 0.97] and 0.76 [0.47, 1.26], respectively) and docetaxel 75 mg/m^2^ (HR [95% CrI]: 0.63 [0.40, 1.02]). T-DXd 5.4 mg/kg showed a notably better ORR when compared with all comparators (ORs ranging from 6.09 [2.26, 18.80] to 21.14 [8.12, 64.59]), except paclitaxel + bevacizumab, for which the OR was still numerically in favor of T-DXd (OR [95% CrI]: 2.09 [0.35, 10.04].

The wide CrIs obtained for the OS HR in the NMA are likely to be driven by the relatively immature OS data for T-DXd, wherein 63.7% (65/102) of patients were censored at the time of the primary analysis. In addition, T-DXd patients had a higher proportion of brain metastases. Patients also had more prior lines of therapy in DESTINY-Lung02 than INTEREST, even after propensity score weighting, suggesting patients in the DESTINY-Lung02 trial were heavily pretreated. Another noticeable difference was that in INTEREST, a greater proportion of patients received subsequent therapies (48%) as compared to DESTINY-Lung02 (32.5%), which likely resulted in an overestimation of the treatment effect of docetaxel compared to T-DXd. Most comparator trials had a higher proportion of patients receiving any subsequent therapy (ranging from 38.2% for the docetaxel arm of IFCT-1103 ULTIMATE to 98.7% for the pemetrexed arm of CTONG0806, with most around 50%) compared to DESTINY-Lung02 (32.5%), which may have further overestimated efficacy for those comparators. These analyses are therefore considered to be conservative, favoring the comparators and may underestimate the significance of the improved treatment efficacy of T-DXd 5.4 mg/kg. The PFS analysis, which was not affected by differences in subsequent therapies, and was based on more mature data (43.1% [44/102] patients with events and 56.9% [58/102] patients censored) from DESTINY-Lung02, shows benefit consistently across all comparisons.

In alignment with the NMA results, in the MAICs, T-DXd 5.4 mg/kg was associated with significantly better PFS compared to all comparators (HRs [95% CI] ranging from 0.23 [0.12, 0.44] to 0.46 [0.25, 0.85]). OS favored T-DXd 5.4 mg/kg for all comparisons (HRs [95% CI] ranging from 0.26 [0.12, 0.57] to 0.64 [0.35, 1.15]) with statistical significance observed versus docetaxel (0.51 [0.27, 0.98]) and paclitaxel + bevacizumab (0.26 [0.12, 0.57]). The MAICs for atezolizumab and ramucirumab + docetaxel were conducted with a weighting based on ITT population characteristics (i.e., mixed histology population) of the comparator trials and therefore should be interpreted with some discretion. Of note, the low ESS found in all MAICs, corresponding to less than 30% of the original DESTINY-Lung02 T-DXd 5.4 mg/kg sample, may indicate insufficient overlap in the patient demographics between T-DXd 5.4 mg/kg and comparator trials, limiting the comparability of the data.

Due to the DESTINY-Lung02 trial design, there is no direct comparison of the efficacy of T-DXd compared to other therapeutic options for individuals with HER2m NSQ a/mNCSLC whose disease has progressed following prior systemic therapies. To address this, this study used the best available evidence and multiple methods following best practice guidelines (41, 42) to indirectly compare the efficacy of T-DXd to other currently available therapies in the population of interest in a comprehensive manner. The ECA utilized IPD to match patient characteristics to the DESTINY02 trial based on available data and connect T-DXd 5.4 mg/kg to the NMA network. There were some remaining discrepancies between the T-DXd 5.4 mg/kg and docetaxel 75 mg/m^2^ treatment arms after propensity score weighting; however, the results from the NMAs using both INTEREST and VITAL ECAs were aligned, suggesting the results are reliable. Moreover, both the NMA and MAIC consistently indicated superior efficacy for T-DXd 5.4 mg/kg, despite some numerical differences were observed in the outputs. In order to assess uncertainty, several sensitivity analyses were conducted and results were consistently aligned with the base case analyses, highlighting the reliability of the conclusions. In particular, the analysis using the longer follow-up data from DESTINY-Lung02 was consistent with the primary analysis, demonstrating that the superior efficacy of T-DXd persists with time. Thus, despite the limitations and potential sources of bias for each method, the similar results across methods and sensitivity analyses lend credence to the conclusions.

There were some limitations in this study. The PHA was rejected for the NMA and MAIC PFS comparisons for pemetrexed and nintedanib + docetaxel and the NMA OS comparison for paclitaxel + bevacizumab. However, due to the star shape of the network, the validity of the results for the other comparisons for the NMA is not impacted. In addition, the ESS was relatively low in the MAICs. Sensitivity analyses using the FAS population were conducted for the MAIC to assess the potential impact of the PHA rejection and provide further insight into the robustness of the results, as the PHA was holding in the FAS analysis. It is also noted that the median follow-up in some trials (CTONG0806, REVEL, DELTA, and LUME-Lung1) (38, 39, 43–45) is relatively short compared to other studies. Therefore, caution should be exercised when interpreting the results for comparison versus nintedanib + docetaxel and pemetrexed. Lastly, the ability to adjust for all prognostic factors and TEMs for the unanchored MAICs was limited as not all factors were available in both DESTINY-Lung02 and comparator trials. Baseline characteristics for the NSQ population were also unavailable for some comparator trials, requiring the use of ITT data as a proxy to still allow for a comparison to be made. Moreover, residual differences in terms of follow-up time, type and number of subsequent treatments, and other unknown factors, between T-DXd and comparator trials cannot always be adjusted for. Despite limitations in the published data, this study employed recommended methods to assess the comparative efficacy of T-DXd and provides evidence suggesting that T-DXd offers improved efficacy for patients with HER2m a/mNSCLC. Comparative safety and tolerability was not assessed in this study and is an important area of future research.

Clinical experts identified the HER2 mutation as a negative prognostic factor, as patients with HER2m NSCLC typically have worse survival outcomes than wild-type HER2 NSCLC (11, 46, 47). However, it was not possible to limit the analyses to HER2m patients due to a lack of information on the presence of this mutation in comparator trials. Given the data limitation on HER2m in published comparator literature, careful assessment of other population characteristics was made to ensure a broadly comparable population among included studies. Comparator trials were included in the analysis only if all patients or a vast majority of them harbored wide-type oncogenic drivers other than HER2m, and results were reported in the NSQ NSCLC population, as this population is more reflective of the patient population in DESTINY-Lung02. Another important consideration given that none of comparators were HER2-targeted regimens, is that the treatment effect is not anticipated to be modified by the expression or absence of HER2 mutation. Considering these two factors, the ITCs presented here are considered representative; however, residual bias may remain due to a lack of data for comparators in the HER2m population. Studies to gather data on other treatments in HER2m patients, for example, from real world data, might address the evidence gap for this patient population and further support treatment recommendations.

T-DXd is currently recommended by ESMO, ASCO, and NCCN and approved by the FDA and EMA for patients with previously treated HER2m a/mNSCLC (19, 22, 27, 28, 48). This study suggested that T-DXd, as the first approved HER2-directed therapy, is associated with better efficacy outcomes in terms of OS, PFS, and ORR when compared to most comparators, supporting the use of T-DXd as an optimal treatment in this NSCLC population. Despite some observed differences in patient characteristics across the trials, such as immaturity of the OS data and differences in the number of prior lines of therapy and subsequent therapies, the treatment effect is very pronounced and is in line with what has been reported for other indications for T-DXd (49, 50). This insight is particularly valuable in the absence of evidence from RCTs.

## Data Availability

Anonymized individual participant data (IPD) on complete studies and applicable supporting clinical study documents may be available upon request at https://vivli.org/. In cases where clinical study data and supporting documents are provided pursuat the Daiichi Sankyo company policies and procedures, Daiichi Sankyo Companies will continue to protect the privacy of comany and clinical study subjects. Details on data sharing criteria and the procedure for requesting access can be found at this web address: https://vivli.org/ourmember/daiichi-sankyo/.
