# Supplementary material for: Comparative efficacy of trastuzumab deruxtecan versus guideline-recommended treatments for 2L+ unresectable locally advanced or metastatic HER2-mutant non-small cell lung cancer: a systematic review and indirect treatment comparison
Source: Front Oncol. 2026 Feb 13;15:1708245. doi: 10.3389/fonc.2025.1708245 (PMC12945754; doi:10.3389/fonc.2025.1708245)
Supplement: Supplementary file 1 [file DataSheet1.docx]

Supplementary Material

# Supplementary Data

## Supplementary Methods

### Systematic Literature Review

The systematic literature review (SLR) conducted to identify relevant clinical trials included adult (≥18 years) patients with NSQ a/mNSCLC harbouring any HER2 gene alteration, or who had wild-type onco-driver genes or an undetermined or mixed mutation status for currently established actionable genetic mutations were included. Interventions and comparators included all second or later-line treatment approved or in development for the treatment of NSCLC or best supportive care, including chemotherapies, targeted therapies, and immunotherapies used in combination or as monotherapies. Electronic searches were conducted on 1^st^ September 2020 in the Embase, Medline, PubMed, Cochrane, and Dare databases. Screening was performed by two independent reviewers, with quality check by a third reviewer. Data extraction was done by one research analyst with a quality check by an independent reviewer. Complementary handsearching was conducted in 2023 to identify new publications for existing studies that were originally included in the 2020 SLR. Studies were then further refined for the ITC based on the criteria outlined in Table 1.

### Generation of ECA

A targeted search was conducted to identify trials that assessed active treatments in patients with NSCLC that would have a comparator arm already included in the network and enable the generation of an ECA. To ensure individual patient data would be accessible, RCTs sponsored by AstraZeneca and publicly available data from Project Data Sphere (1) were considered. Seven AstraZeneca-sponsored trials were identified that assessed efficacy outcomes in 2^nd^ line and later standard of care in stage IIIB/IV NSCLC. The INTEREST trial (2), which used docetaxel in the control arm, was preferred as it treated patients with second or subsequent lines of therapy. In addition, ten trials in NSCLC were identified in the Project Data Sphere database; three were excluded due to patients being treated with first-line therapy, another three were excluded as patients had stage III/IIIA at inclusion, and two were excluded because the trial assessed the maintenance treatment of non-progressing patients. Of the remaining two trials, VITAL (3) was chosen as it published detailed patient characteristics and efficacy data for the comparator arm (docetaxel).

Propensity score weighting was used to create an ECA with comparable prognostic factors to patients enrolled in DESTINY-Lung02. This approach estimated the average treatment effect among the treated (ATT), maximizing the amount of information used from the trials. The propensity scores were generated using a logistic regression that estimated the conditional probability of a patient being treated with T-DXd 5.4 mg/kg versus the treatment from the external arm based on their baseline characteristics. The propensity scores were then used to estimate a weight for each patient to make the treatment groups from INTEREST and VITAL more similar to DESTINY-Lung02. The ECA was assessed by reviewing the effective sample size (ESS) and the distribution of the propensity score weights by treatment arm. Descriptive characteristics were also assessed for the reweighted populations to determine whether substantial differences remained between the groups. The covariates included in the logistic regression are those listed in the methods (section 2.1). If there were more than 20% of patients with missing values for a covariate, the covariate was not used for the propensity score.

The baseline characteristics before and after weighting are provided in Table S2. The weights distribution are illustrated in Figure S3. Before adjustment, the proportions of women and non-smokers were significantly lower than in INTEREST (40.2% and 24.0%) and VITAL (37.7% and 32.7%) compared to the T-DXd 5.4 mg/kg arm from DESTINY-Lung02 (64.8% and 58.0%). In addition, patients enrolled in DESTINY-Lung02 had received significantly more previous regimens (≥2 regimens: 70.5%), and a higher proportion of patients had brain metastases (38.6%). Of the 102 patients from the T-DXd 5.4 mg/kg arm of the DESTINY-Lung02 trial, eleven patients were removed from the anonymization process and 3 were removed due to missing demographic information, leaving 88 patients included in the analysis for weighting. After adjustment through propensity score weighting, the ESS was 72.5 and 88.0 for docetaxel 75 mg/m^2^ assessed in INTEREST and VITAL, respectively.

Given the differences in the proportion of patients with ≥2 prior therapies between VITAL and DESTINY-Lung02 (1.4% vs. 70.5%, after weighting), which resulted in imbalances in patients’ weighting, INTEREST was determined to be the most suitable trial to construct the ECA, with VITAL considered in a sensitivity analysis.

### NMA

The proportional hazard assumption (PHA) was investigated for all studies included in the PFS and OS networks. The Kaplan–Meier (KM) curves were digitized for comparator trials and the individual patient data (IPD) were reconstructed based on the Guyot algorithm (4). Assessment of the PHA was based on the visual inspection of the KM curves, log-cumulative hazards plots, the Schoenfeld residuals, and the Grambsch and Therneau test (5–8). To assess statistical heterogeneity in the network, the Cochran’s Q test was conducted, and the I^2^ statistics were calculated (9). Heterogeneity was suspected if the Cochran’s Q test was significant with a significance level of 10%, or I^2^ was higher than 50%. Inconsistency was assessed by comparing the direct versus indirect evidence within each loop of the network using the Bucher approach (10). If heterogeneity or inconsistency was suspected, trials driving the heterogeneity were excluded from the base case and were included in a sensitivity analysis.

Given that only one trial was used in each comparison, the random-effect (RE) model was not expected to converge; therefore, the fixed-effect (FE) model was selected. The NMA was performed with WinBUGS V1.4 using the Markov Chain Monte Carlo (MCMC) simulation method. For the model estimations, three chains were simulated, and 20,000 iterations were used as burn-in, followed by 20,000 iterations to monitor the parameters for the FE model. Convergence was confirmed by evaluation of the three chains and Brooks-Gelman-Rubin plots.

### MAIC

The unanchored MAICs were implemented by first estimating the weights associated with each individual patient treated with T-DXd 5.4 mg/kg through the generation of a logistic regression model. Then, relevant statistical analyses were generated using the unweighted and weighted data from the DESTINY-Lung02 T-DXd 5.4 mg/kg arm and the reconstructed IPD data from the comparators’ trial. To ensure the validity of the analysis, the distribution of weights was compared, ESS was reviewed, and trial baseline characteristics were assessed for imbalances. The validity of the reconstructed IPD was assessed based on the median time to event reported in publications versus the reconstructed one with the associated 95% CI. The HR between the T-DXd 5.4 mg/kg arm outcome data and the comparator outcome data was estimated using a weighted Cox proportional hazards model based on reconstructed IPD and assessing the PHA (using the same approaches as for the NMA) between the reconstructed IPD obtained through the Guyot algorithm (4) and the weighted T-DXd 5.4 mg/kg arm.

## Supplementary Results

### PFS NMA sensitivity analysis with all studies

SIGN and CTONG0806 were excluded from the base-case PFS network due to inconsistency in the loops for gefitinib, pemetrexed, erlotinib, and docetaxel 75 mg/m^2^. A sensitivity analysis was performed on the overall network to assess the robustness of the base case scenario. The biggest numerical differences between the sensitivity analysis and base case were found for docetaxel 60 mg/m^2^ (HR [95% CrI]: 0.22 [0.13, 0.39] versus 0.18 [0.10, 0.31], respectively) and pemetrexed (0.22 [0.13, 0.37] versus 0.15 [0.09, 0.26]) (Table S 4). Overall, conclusions remained the same, with T-DXd showing a notably better PFS than all comparators.

### MAIC sensitivity analysis on the full analysis set

A sensitivity analysis was performed for each MAIC on the full analysis set of DESTINY-Lung02, without restricting the population to match the comparator trial. The sensitivity analyses also included alternative trials not included in the base-case analyses for docetaxel (IFCT-1103 ULTIMATE) and atezolizumab (POPLAR). The results from the full analysis set sensitivity analysis and restricted population base-case analysis were similar, with more results reaching significance for OS (Table S 7). As with the base-case MAICs, T-DXd was found to have a significantly longer PFS than all comparators (HRs [95% CI] ranging from 0.17 [0.11, 0.29] versus nintedanib + docetaxel to 0.36 [0.22, 0.57] versus nivolumab). For OS, T-DXd was found to have a significantly longer OS than docetaxel (HR [95% CI]: 0.51 [0.27, 0.94]), ramucirumab + docetaxel (0.42 [0.27, 0.67]), bevacizumab + paclitaxel (0.32 [0.17, 0.61]), and nintedanib + docetaxel (0.56 [0.36, 0.88]).

### MAIC sensitivity analysis using DESTINY-Lung02 25 August 2023 data cut-off

A sensitivity analysis was conducted using the later data cut-off for DESTINY-Lung02. The sensitivity analyses also included alternative trials not included in the base-case analyses for docetaxel (IFCT-1103 ULTIMATE) and atezolizumab (POPLAR). The results were consistent with those based on the primary data cut-off. As in the primary data cut-off analyses, T-DXd was found to have significantly longer PFS than all comparators, with similar HR point estimates and narrower CIs (Figure S 2). Results for OS were also similar between the analyses, with the narrower CIs for the later data cut-off meaning significance was reached for the comparisons versus nivolumab (HR [95% CI]: 0.56 [0.30, 1.04] for primary data cut-off versus 0.51 [0.29, 0.90] for later data cut-off) and ramucirumab + docetaxel (0.54 [0.28, 1.04] for primary data cut-off versus 0.55 [0.32, 0.97] for later data cut-off) (Figure S 2).

# Supplementary Figures and Tables

## Supplementary Figures


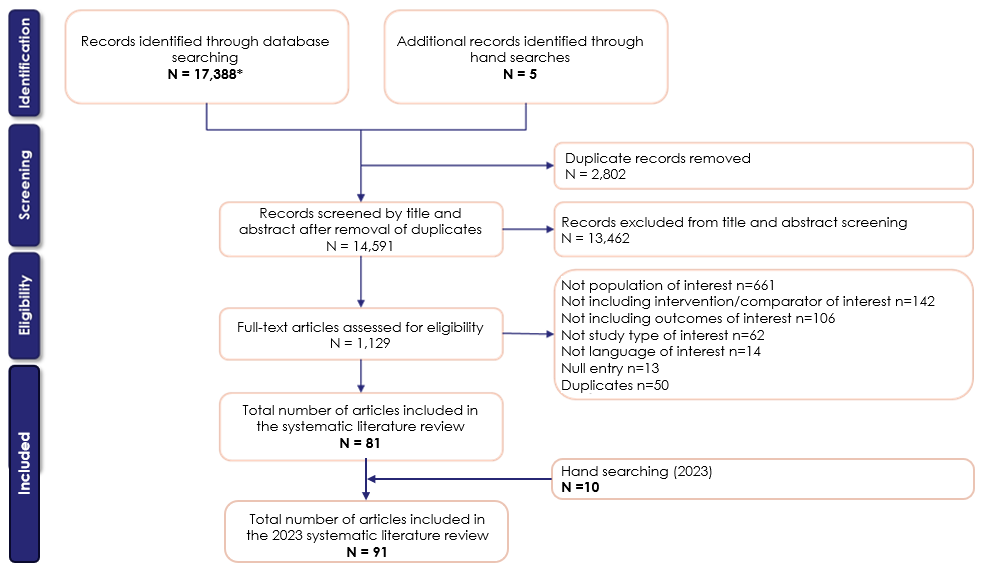
Supplementary Figure S1. PRISMA diagram for the SLR for studies including confirmed non-squamous NSCLC patients *Embase [N = 9,177] + PubMed [N = 6,506] + Cochrane [N = 1,326] + DARE [N = 379]. SLR: systematic literature review

**
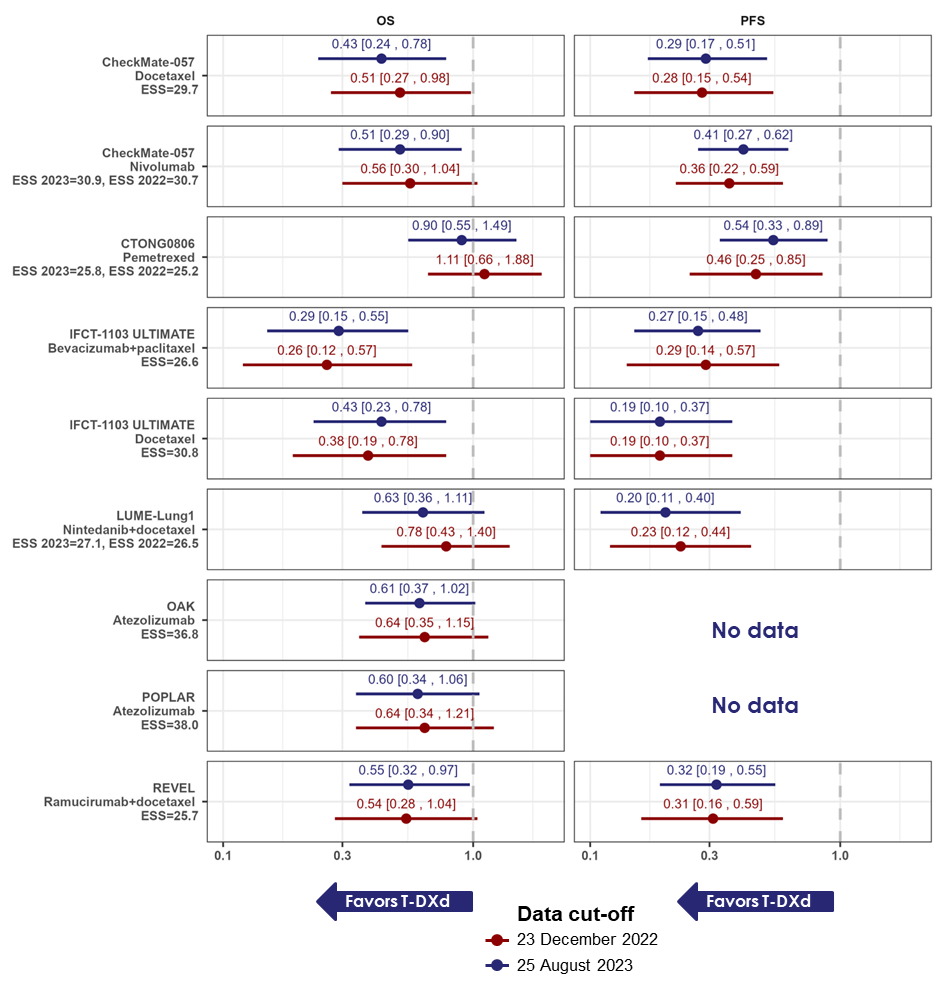
**

Supplementary Figure S2. Plot of PFS and OS results from the MAIC comparing DESTINY-Lung02 December 2022 data cut-off versus August 2023. The December 2022 data cut-off is in red and the August 2023 data cut-off is in blue. Both analyses indicated results in favor of T-DXd. Note: When updating the MAICs, it was noted that one patient was identified as receiving only one prior line of therapy, having previously been coded as receiving two prior lines of therapy. This was as a result of receiving maintenance therapy and not a separate treatment line. This update had very small impact on the estimated patient weights for some comparisons (as seen from the slightly changed ESS). Abbreviations: CI: confidence interval; ESS: effective sample size; HR: hazard ratio; MAIC: matching-adjusted indirect comparison; OS: overall survival; PFS: progression-free survival.


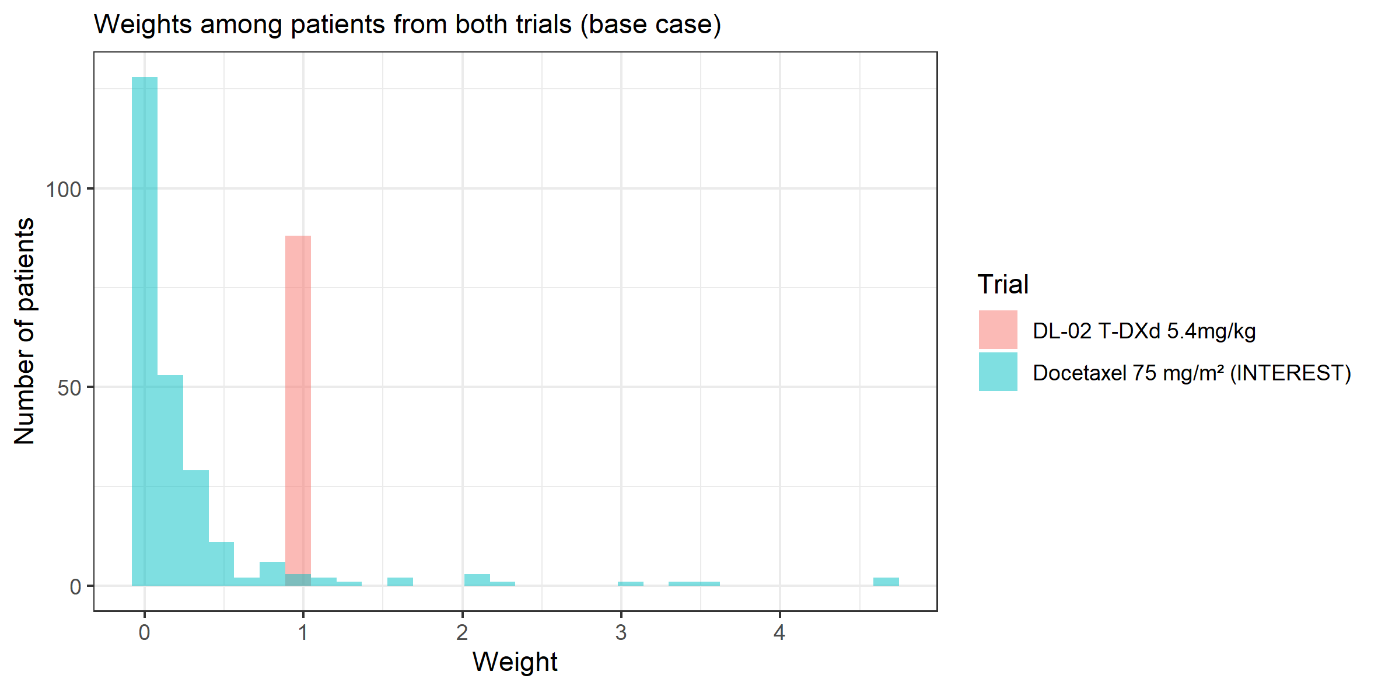


A.


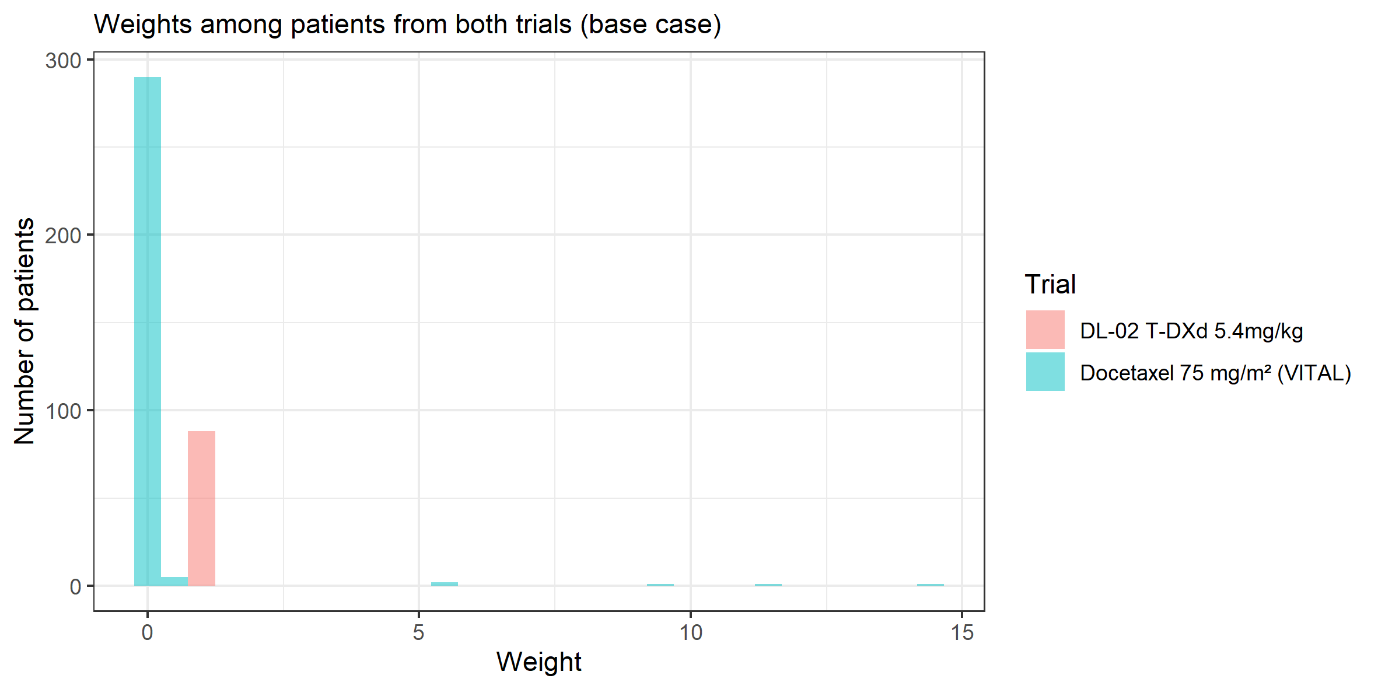


B.

**Supplementary Figure S3. Weights distribution for the docetaxel 75 mg/m^2^ treatment arm of INTEREST (A) and VITAL (B) and the T-DXd 5.4 mg/kg treatment arm of DESTINY-Lung02**.

## Supplementary Tables

## Supplementary Table S1. Summary of trials excluded from analyses

| Comparator | Trial | NMA | | | MAIC | |
| --- | --- | --- | --- | --- | --- | --- |
|  |  | **PFS** | **OS** | **ORR** | **PFS** | **OS** |
| Docetaxel 75 mg/m² | PROLUNG (11) | Trial excluded given irrelevant end node | | | Lack of baseline for NSQ + lack of outcomes for NSQ | |
| Docetaxel 60 mg/m^2^ | WJOG5910L (12) | Trial excluded given irrelevant end node | | | Treatment not of interest for MAIC | |
| Nivolumab | NCT02175017 (13) | Single-arm trial | | | CheckMate057 preferred based on study type | |
| Nintedanib + docetaxel | NCT02392455 (VARGARDO) (14) | Single-arm trial | | | LUME-Lung1 preferred based on population | |
| Pemetrexed | NCT00550173 (15) | Trial excluded based on population heterogeneity (non-smokers only) | | | | |
|  | LUME-Lung2 (16) | Trial excluded given irrelevant end node | | | CTONG0806 preferred vs. pemetrexed due to inclusion of EGFR-mutated patients | |
|  | Hattori 2014 | Single-arm trial | | | CTONG0806 preferred based on study type | |
|  | Kim 2010 | Single-arm trial | | | Lack of baseline for NSQ + lack of KM curves | |
|  | NCT00380718 (17) | Single-arm trial | | | Lack of baseline for NSQ + lack of KM curves | |
| Cisplatin | Du 2013 (18) | Trial disconnected from the network | | | Lack of KM curves | |

Abbreviations: KM: Kaplan-Meier; MAIC: matching-adjusted indirect comparison; NMA: network meta-analysis; NSQ: non-squamous; ORR: overall response rate; OS: overall survival; PFS: progression-free survival.

Supplementary Table S2. Baseline characteristics comparing patients in the T-DXd 5.4 mg/kg treatment arm of DESTINY-Lung02 to the docetaxel 75 mg/m^2^ treatment arm of INTEREST (base case analysis) and VITAL before and after weighting (sensitivity analysis)

|  | T-DXd 5.4 mg/kg (N=88) | INTEREST | | | | VITAL | | | |
| --- | --- | --- | --- | --- | --- | --- | --- | --- | --- |
|  |  | **Docetaxel 75 mg/m^2^* prior weighting (N=246)** | **P-value prior weighting vs. DESTINY-Lung02** | **Docetaxel 75 mg/m^2^* after weighting (ESS=72.5)** | **P-value after weighting vs. DESTINY-Lung02** | **Docetaxel 75 mg/m^2^* prior weighting (N=300)** | **P-value prior weighting vs. DESTINY-Lung02** | **Docetaxel 75 mg/m^2^* after weighting (ESS=88.0)** | **P-value after weighting vs. DESTINY-Lung02** |
| **Age category** | | | | | | | | | |
| ≤65 | 55 (62.5%) | 186 (75.6%) | **<0.05** | 43 (59.4%) | 0.81 | 213 (71.0%) | 0.17 | 54.9 (62.4%) | >0.99 |
| >65 | 33 (37.5%) | 60 (24.4%) |  | 29.4 (40.6%) |  | 87 (29.0%) |  | 33.1 (37.6%) |  |
| **Sex** | | | | | | | | | |
| Female | 57 (64.8%) | 99 (40.2%) | **<0.05** | 44.4 (61.2%) | 0.77 | 113 (37.7%) | **<0.05** | 57 (64.7%) | >0.99 |
| Male | 31 (35.2%) | 147 (59.8%) |  | 28.1 (38.8%) |  | 187 (62.3%) |  | 31.1 (35.3%) |  |
| **Disease stage^†^** | | | | | | | | | |
| Metastatic | 88 (100%) | 246 (100%) | NA | 72.5 (100%) | NA | 300 (100%) | NA | 88.0 (100%) | NA |
| **Histology^†^** | | | | | | | | | |
| Non-squamous | 87 (98.9%) | 246 (100%) | 0.26 | 72.5 (100%) | >0.99 | 300 (100%) | 0.23 | 88.0 (100%) | 1 |
| Squamous | 1 (1.1%) | 0 (0%) |  | 0 (0%) |  | 0 (0%) |  | 0 (0%) |  |
| **Smoking status** | | | | | | | | | |
| Current smoker | 0 (0%) | 0 (0%) | **<0.05** | 0 (0%) | 0.74 | 0 (0%) | 0.57 | 0 (0%) | 1 |
| Former smoker | 37 (42.0%) | 187 (76.0%) |  | 33.3 (46.0%) |  | 202 (67.3%) |  | 37 (42.0%) |  |
| Never smoker | 51 (58.0%) | 59 (24.0%) |  | 39.2 (54.0%) |  | 98 (32.7%) |  | 51.1 (58.0%) |  |
| **ECOG PS** | | | | | | | | | |
| 0 | 25 (28.4%) | 74 (30.1%) | 0.87 | 22.6 (31.3%) | 0.83 | 97 (32.3%) | 0.65 | 23.8 (27.0%) | 0.97 |
| 1 | 63 (71.6%) | 172 (69.9%) |  | 49.8 (68.7%) |  | 203 (67.7%) |  | 64.3 (73.0%) |  |
| **Brain metastases** | | | | | | | | | |
| No | 54 (61.4%) | 227 (92.3%) | **<0.05** | 60 (82.8%) | **<0.05** |  |  |  |  |
| Yes | 34 (38.6%) | 19 (7.7%) |  | 12.5 (17.2%) |  |  |  |  |  |
| **Liver metastases** | | | | | | | | | |
| No | 70 (79.5%) | 196 (79.7%) | 1 | 59.3 (81.8%) | 0.88 |  |  |  |  |
| Yes | 18 (20.5%) | 50 (20.3%) |  | 13.2 (18.2%) |  |  |  |  |  |
| **Number of previous regimens^†^** | | | | | | | | | |
| One previous regimen | 26 (29.5%) | 210 (85.4%) | **<0.05** | 24.6 (33.9%) | 0.68 | 295 (98.3%) | **<0.05** | 86.8 (98.6%) | **<0.05** |
| At least 2 previous regimens | 62 (70.5%) | 36 (14.6%) |  | 47.9 (66.1%) |  | 5 (1.7%) |  | 1.2 (1.4%) |  |
| **Tumour diameter^†^** | | | | | | | | | |
| Mean (SD) | 37.7 (21.8) | 40.5 (22.0) | 0.34 | 39.3 (21.7) | 0.66 | 40.6 (24.9) | 0.32 | 38.7 (23.1) | 0.79 |
| Median (Q1-Q3) | 32  (21.0-51.0) | 36.5  (24.0-51.0) |  | 35  (19.7-60.0) |  | 36.8  (22.2-50.0) |  | 35.2  (23.0-50.0) |  |

*Assessed in INTEREST/VITAL

**^†^**Variables not used in the weighting process

Abbreviations: ECOG PS: Eastern Cooperative Oncology Group performance status; ESS: effective sample size; NA: not applicable; Q1: first quartile; Q3: third quartile; SD: standard deviation; T-DXd: trastuzumab deruxtecan

Supplementary Table S3. Inconsistency assessment in PFS network

| Comparison | Trial/Analysis | HR [95% CI] | Z statistic | P-value |
| --- | --- | --- | --- | --- |
| Docetaxel 75 mg/m^2^ vs. gefitinib | SIGN | 1.06 [0.72, 1.56] | 2.75 | **0.01** |
| Pemetrexed vs. Gefitinib | CTONG0806 | 0.53 [0.38, 0.75] |  |  |
| Erlotinib vs. Pemetrexed | HORG | 1.03 [0.79, 1.33] |  |  |
| Docetaxel 75 mg/m² vs. erlotinib | Indirect | 1.94 [1.09, 3.46] |  |  |
|  | Direct (TAILOR) | 0.76 [0.54, 1.05] |  |  |

Abbreviations: CI: confidence interval; HR: hazard ratio; PFS: progression-free survival.

Supplementary Table S4. Conclusion of the PHA assessment for the NMA

| **Comparator** | **Trial** | **OS** | **PFS** |
| --- | --- | --- | --- |
| **Atezolizumab** | OAK (19) | PHA is not rejected from 4 months |  |
|  | POPLAR (20) | PHA is not rejected from 8 months |  |
| **Nivolumab** | CheckMate 057 (21) | PHA is not rejected from 8 months | PHA is not rejected from 7 months |
|  | CheckMate 078 (22) | PHA is not rejected from 8 months | PHA is not rejected from 7 months |
| **Ramucirumab + docetaxel** | REVEL (23) |  |  |
| **Paclitaxel + bevacizumab** | IFCT-1103 ULTIMATE (24) |  |  |
| **Gefitinib** | SIGN (25) |  |  |
| **Nintedanib + docetaxel** | LUME-Lung 1 | PHA is not rejected from 6 months |  |
| **Pemetrexed** | CTONG0806 (26) |  |  |

: KM not available for non-squamous patients, : Study not included in network, : PHA not rejected, : PHA rejected over during the first months of the study, : PHA rejected

Abbreviations: KM: Kaplan-Meier; OS: overall survival; PFS: progression-free survival; PHA: proportional hazard assumption.

Supplementary Table S5. Estimated HRs of T-DXd 5.4 mg/kg versus comparators in the base case excluding SIGN and CTONG0806 and the inconsistency sensitivity analysis on the overall network

| Comparators | HR [95% CrI] | |
| --- | --- | --- |
|  | **Base case without SIGN** (25) **and CTONG0806** (26) | **Inconsistency sensitivity analysis on the overall network** |
| Docetaxel 75 mg/m^2^ | 0.20 [0.14, 0.30] | 0.20 [0.14, 0.30] |
| Docetaxel 60 mg/m^2^ | 0.18 [0.10, 0.31] | 0.22 [0.13, 0.39] |
| Nivolumab | 0.23 [0.15, 0.34] | 0.22 [0.15, 0.34] |
| Pemetrexed | 0.15 [0.09, 0.26] | 0.22 [0.13, 0.37] |
| Paclitaxel + bevacizumab | 0.33 [0.20, 0.56] | 0.33 [0.20, 0.55] |
| Ramucirumab + docetaxel | 0.27 [0.17, 0.40] | 0.26 [0.17, 0.40] |
| Pembrolizumab | 0.24 [0.15, 0.36] | 0.23 [0.15, 0.36] |
| Nintedanib + docetaxel | 0.26 [0.17, 0.41] | 0.26 [0.17, 0.41] |

Abbreviations: CrI: credible interval; HR: hazard ratio; T-DXd: trastuzumab deruxtecan.

Supplementary Table S6. Conclusion of PHA assessment for MAIC

| **Comparator** | **Trials** | **OS** | **PFS** |
| --- | --- | --- | --- |
| **Docetaxel** | CheckMate 057 (21) | PHA is not rejected from 3 months |  |
|  | IFCT-1103 ULTIMATE (24) | PHA is not rejected until 18 months |  |
| **Nivolumab** | CheckMate 057 (21) | PHA is not rejected from 3 months |  |
| **Bevacizumab + paclitaxel** | IFCT-1103 ULTIMATE (24) | PHA is not rejected from 3 months until 19 months | PHA is not rejected from 2 months |
| **Pemetrexed** | CTONG0806 (26) |  |  |
| **Nintedanib + docetaxel** | LUME-Lung1 (27) |  |  |
| **Atezolizumab** | OAK (19) | PHA is not rejected from 3 months until 19 months |  |
| **Atezolizumab** | POPLAR (20) | PHA is not rejected from 3 months until 19 months |  |
| **Ramucirumab + docetaxel** | REVEL (23) | PHA is not rejected from 3 months until 19 months |  |
| **Pemetrexed** | CTONG0806 (26) | PHA is not rejected until 15 months |  |
| **Nintedanib + docetaxel** | LUME-Lung1 (27) |  |  |

: KM not available for non-squamous patients, : Study not included in network, : PHA not rejected, : PHA rejected over during the first months of the study, : PHA rejected

Abbreviations: KM: Kaplan-Meier curves; MAIC: matching-adjusted indirect comparison; PHA: proportional hazard assumption.

Supplementary Table S7. Summary of sensitivity analysis MAIC results using DESTINY-Lung02 full-analysis set

| Comparator | Trial used to inform comparison | Outcome | Base case analysis | Sensitivity analysis | ESS (% of initial sample) | Weighted HR [95% CI]  T-DXd vs. comparator |
| --- | --- | --- | --- | --- | --- | --- |
| Docetaxel | CheckMate 057 (21) | PFS | Apply inclusion criteria of comparators’ trial to T-DXd patients before adjustment, i.e., restricted to 2^nd^ or 3^rd^ line patients and reweighted | Without restriction, i.e., based on DESTINY-Lung02 full-analysis set | 31.3 (30.5%) | **0.28 [0.15, 0.51]** |
|  |  | OS |  |  |  | **0.51 [0.27, 0.94]** |
|  | IFCT-1103 ULTIMATE (24) | PFS | Apply inclusion criteria of comparators’ trial to T-DXd patients before adjustment, i.e., restricted to 2^nd^ or 3^rd^ line patients and reweighted | Without restriction, i.e., based on DESTINY-Lung02 full-analysis set | 38.1 (37.4%) | **0.19 [0.11, 0.35]** |
|  |  | OS |  |  |  | **0.46 [0.25, 0.85]** |
| Ramucirumab + docetaxel | REVEL (23) (using ITT as proxy) | PFS | Apply inclusion criteria of comparators’ trial to T-DXd patients before adjustment, i.e., restricted to 2^nd^ line patients and reweighted | Without restriction, i.e., based on DESTINY-Lung02 full-analysis set | 57.7 (56.6%) | ***0.30 [0.20, 0.46]*** |
|  |  | OS |  |  |  | ***0.42 [0.27, 0.67]*** |
| Nivolumab | CheckMate 057 (21) | PFS | Apply inclusion criteria of comparators’ trial to T-DXd patients before adjustment, i.e., restricted to 2^nd^ or 3^rd^ line patients and reweighted | Without restriction, i.e., based on DESTINY-Lung02 full-analysis set | 32.2 (31.6%) | **0.36 [0.22, 0.57]** |
|  |  | OS |  |  |  | 0.56 [0.31, 1.01] |
| Atezolizumab | OAK (19) (using ITT as proxy) | OS | Apply inclusion criteria of comparators’ trial to T-DXd patients before adjustment, i.e., restricted to 2^nd^ or 3^rd^ line patients and reweighted | Without restriction, i.e., based on DESTINY-Lung02 full-analysis set | 41.3 (40.5%) | *0.67 [0.40, 1.13]* |
|  | POPLAR (20) (using ITT as proxy) | OS | Apply inclusion criteria of comparators’ trial to T-DXd patients before adjustment, i.e., restricted to 2^nd^ or 3^rd^ line patients and reweighted | Without restriction, i.e., based on DESTINY-Lung02 full-analysis set | 48.7 (47.7%) | *0.69 [0.40, 1.20]* |
| Bevacizumab + paclitaxel | IFCT-1103 ULTIMATE (24) | PFS | Apply inclusion criteria of comparators’ trial to T-DXd patients before adjustment, i.e., restricted to 2^nd^ or 3^rd^ line patients and reweighted | Without restriction, i.e., based on DESTINY-Lung02 full-analysis set | 35.5 (34.8%) | **0.30 [0.17, 0.54]** |
|  |  | OS |  |  |  | **0.32 [0.17, 0.61]** |
| Nintedanib + docetaxel | LUME-Lung1 (27) | PFS | Apply inclusion criteria of comparators’ trial to T-DXd patients before adjustment, i.e., restricted to 2^nd^ line patients but not reweighted | Without restriction, i.e., based on DESTINY-Lung02 full-analysis set | 54.6 (53.5%) | **0.17 [0.11, 0.29]** |
|  |  | OS |  |  |  | **0.56 [0.36, 0.88]** |
| Pemetrexed | CTONG0806 (26) | PFS | Apply inclusion criteria of comparators’ trial to T-DXd patients before adjustment, i.e., restricted to 2^nd^ line patients but not reweighted | Without restriction, i.e., based on DESTINY-Lung02 full-analysis set | 64.1 (62.8%) | **0.50 [0.33, 0.76]** |
|  |  | OS |  |  |  | 0.79 [0.49, 1.26] |

**Bold** font indicates significant benefit with T-DXd; *italic* font indicates an adjustment on ITT characteristics to overcome lack of non-squamous baseline characteristics

Abbreviations: CI: confidence interval; ESS: effective sample size; HR: hazard ratio; MAIC: Matching Adjusted Indirect Comparison; OS: overall survival; PFS: progression-free survival; T-DXd: trastuzumab deruxtecan.

# References

1. Project Data Sphere [Internet]. [cited 2025 Jan 20]. Available from: https://data.projectdatasphere.org/projectdatasphere/html/access

2. Kim ES, Hirsh V, Mok T, Socinski MA, Gervais R, Wu YL, et al. Gefitinib versus docetaxel in previously treated non-small-cell lung cancer (INTEREST): a randomised phase III trial. The Lancet. 2008 Nov 22;372(9652):1809–18.

3. Ramlau R, Gorbunova V, Ciuleanu TE, Novello S, Ozguroglu M, Goksel T, et al. Aflibercept and Docetaxel Versus Docetaxel Alone After Platinum Failure in Patients With Advanced or Metastatic Non–Small-Cell Lung Cancer: A Randomized, Controlled Phase III Trial. JCO. 2012 Oct 10;30(29):3640–7.

4. Guyot P, Ades A, Ouwens MJ, Welton NJ. Enhanced secondary analysis of survival data: reconstructing the data from published Kaplan-Meier survival curves. BMC Med Res Methodol. 2012 Feb 1;12(1):9.

5. Schoenfeld D. Partial residuals for the proportional hazards regression model. Biometrika. 1982;69(1):239-241.

6. Kuitunen I, Ponkilainen VT, Uimonen MM, Eskelinen A, Reito A. Testing the proportional hazards assumption in cox regression and dealing with possible non-proportionality in total joint arthroplasty research: methodological perspectives and review. BMC Musculoskelet Disord. 2021;22(1):489.

7. Grambsch PM, Therneau TM. Proportional Hazards Tests and Diagnostics Based on Weighted Residuals. Biometrika. 1994;81(3.

8. In J, Lee DK. Survival analysis: part II - applied clinical data analysis. Korean J Anesthesiol. 2019;72(5):441-457.

9. Higgins JPT, Thompson SG. Quantifying heterogeneity in a meta-analysis. Stat Med. 2002 June 15;21(11):1539–58.

10. Bucher HC, Guyatt GH, Griffith LE, Walter SD. The results of direct and indirect treatment comparisons in meta-analysis of randomized controlled trials. J Clin Epidemiol. 1997 June;50(6):683–91.

11. Arrieta O, Barrón F, Ramírez-Tirado LA, Zatarain-Barrón ZL, Cardona AF, Díaz-García D, et al. Efficacy and Safety of Pembrolizumab Plus Docetaxel vs Docetaxel Alone in Patients With Previously Treated Advanced Non–Small Cell Lung Cancer: The PROLUNG Phase 2 Randomized Clinical Trial. JAMA Oncology. 2020 June 1;6(6):856–64.

12. Takeda M, Okamoto I, Yamanaka T, Nakagawa K, Nakanishi Y. Impact of treatment with bevacizumab beyond disease progression: a randomized phase II study of docetaxel with or without bevacizumab after platinum-based chemotherapy plus bevacizumab in patients with advanced nonsquamous non–small cell lung cancer (WJOG 5910L). BMC Cancer. 2012 Aug 1;12(1):327.

13. Cho EK, Kang JH, Han JY, Lee JS, Kim DW, Kim SW, et al. Phase II study of nivolumab in patients with advanced non-small cell lung cancer (NSCLC) in Korea. Annals of Oncology. 2017 Apr 1;28:ii41.

14. Grohé C, Wehler T, Dechow T, Henschke S, Schuette W, Dittrich I, et al. Nintedanib plus docetaxel after progression on first-line immunochemotherapy in patients with lung adenocarcinoma: Cohort C of the non-interventional study, VARGADO. Transl Lung Cancer Res. 2022 Oct;11(10):2010–21.

15. Lee DH, Lee JS, Kim SW, Rodrigues-Pereira J, Han B, Song XQ, et al. Three-arm randomised controlled phase 2 study comparing pemetrexed and erlotinib to either pemetrexed or erlotinib alone as second-line treatment for never-smokers with non-squamous non-small cell lung cancer. European Journal of Cancer. 2013 Oct 1;49(15):3111–21.

16. Hanna NH, Kaiser R, Sullivan RN, Aren OR, Ahn MJ, Tiangco B, et al. Lume-lung 2: A multicenter, randomized, double-blind, phase III study of nintedanib plus pemetrexed versus placebo plus pemetrexed in patients with advanced nonsquamous non-small cell lung cancer (NSCLC) after failure of first-line chemotherapy. JCO. 2013 May 20;31(15_suppl):8034–8034.

17. Open-Label Single-Arm Phase IV Study of Pemetrexed in Taiwanese Patients With Advanced Non-Small Cell Lung Cancer Who Have Had Prior Chemotherapy [Internet]. clinicaltrials.gov; 2010 Nov [cited 2025 Jan 24]. Report No.: NCT00380718. Available from: https://clinicaltrials.gov/study/NCT00380718

18. Du N, Li X, Li F, Zhao H, Fan Z, Ma J, et al. Intrapleural combination therapy with bevacizumab and cisplatin for non-small cell lung cancer‑mediated malignant pleural effusion. Oncology Reports. 2013 June 1;29(6):2332–40.

19. Rittmeyer A, Barlesi F, Waterkamp D, Park K, Ciardiello F, Pawel J von, et al. Atezolizumab versus docetaxel in patients with previously treated non-small-cell lung cancer (OAK): a phase 3, open-label, multicentre randomised controlled trial. The Lancet. 2017 Jan 21;389(10066):255–65.

20. Fehrenbacher L, Spira A, Ballinger M, Kowanetz M, Vansteenkiste J, Mazieres J, et al. Atezolizumab versus docetaxel for patients with previously treated non-small-cell lung cancer (POPLAR): a multicentre, open-label, phase 2 randomised controlled trial. The Lancet. 2016 Apr 30;387(10030):1837–46.

21. Borghaei H, Paz-Ares L, Horn L, Spigel DR, Steins M, Ready NE, et al. Nivolumab versus Docetaxel in Advanced Nonsquamous Non–Small-Cell Lung Cancer. New England Journal of Medicine. 2015 Oct 22;373(17):1627–39.

22. Wu YL, Lu S, Cheng Y, Zhou C, Wang J, Mok T, et al. Nivolumab Versus Docetaxel in a Predominantly Chinese Patient Population With Previously Treated Advanced NSCLC: CheckMate 078 Randomized Phase III Clinical Trial. Journal of Thoracic Oncology. 2019 May 1;14(5):867–75.

23. Garon EB, Ciuleanu TE, Arrieta O, Prabhash K, Syrigos KN, Goksel T, et al. Ramucirumab plus docetaxel versus placebo plus docetaxel for second-line treatment of stage IV non-small-cell lung cancer after disease progression on platinum-based therapy (REVEL): a multicentre, double-blind, randomised phase 3 trial. The Lancet. 2014 Aug 23;384(9944):665–73.

24. Cortot AB, Audigier-Valette C, Molinier O, Le Moulec S, Barlesi F, Zalcman G, et al. Weekly paclitaxel plus bevacizumab versus docetaxel as second- or third-line treatment in advanced non-squamous non–small-cell lung cancer: Results of the IFCT-1103 ULTIMATE study. European Journal of Cancer. 2020 May 1;131:27–36.

25. Cufer T, Vrdoljak E, Gaafar R, Erensoy I, Pemberton K, Group on behalf of the S study. Phase II, open-label, randomized study (SIGN) of single-agent gefitinib (IRESSA) or docetaxel as second-line therapy in patients with advanced (stage IIIb or IV) non-small-cell lung cancer. Anti-Cancer Drugs. 2006 Apr;17(4):401.

26. Zhou Q, Cheng Y, Yang JJ, Zhao MF, Zhang L, Zhang XC, et al. Pemetrexed versus gefitinib as a second-line treatment in advanced nonsquamous nonsmall-cell lung cancer patients harboring wild-type *EGFR* (CTONG0806): a multicenter randomized trial†. Annals of Oncology. 2014 Dec 1;25(12):2385–91.

27. Reck M, Kaiser R, Mellemgaard A, Douillard JY, Orlov S, Krzakowski M, et al. Docetaxel plus nintedanib versus docetaxel plus placebo in patients with previously treated non-small-cell lung cancer (LUME-Lung 1): a phase 3, double-blind, randomised controlled trial. The Lancet Oncology. 2014 Feb 1;15(2):143–55.
